# Jon Fosse: „Melancholie“ – eine Lektüre aus psychiatrischer Sicht

**DOI:** 10.1007/s00115-024-01623-7

**Published:** 2024-02-16

**Authors:** Katharina Domschke, Dieter Ebert

**Affiliations:** Klinik für Psychiatrie und Psychotherapie, Medizinische Fakultät, Universitätsklinikum Freiburg, Albert-Ludwigs-Universität Freiburg, Hauptstr. 5, 79104 Freiburg, Deutschland

Der Nobelpreis für Literatur 2023 wurde Jon Fosse (*1959), einem norwegischen Schriftsteller verliehen. Eines seiner Hauptwerke trägt den Titel „Melancholie“ [3], der den psychiatrisch interessierten Leser aufhorchen lässt. Eingebettet in kunsthistorisch wohl korrekte biografische Details schildert Jon Fosse hier letztlich die psychiatrische Krankengeschichte des norwegischen Landschaftsmalers Lars Hertervig (*16.02.1830 in Borgøy, Norwegen, ✝ 06.01.1902 in Stavanger, Norwegen).

Im ersten Teil des Romans, „Melancholie I“ begegnet der Leser Lars Hertervig an einem Nachmittag im Spätherbst 1853 in einem violetten Samtanzug auf seinem Bett liegend in einem bei der Witwe Winckelmann angemieteten Zimmer in der Jägerhofstraße in Düsseldorf. Er ist Student bei den norwegischen Landschaftsmalern Hans F. Gude und Erik Bodom an der Kunstakademie Düsseldorf. Nur einige wenige Fußminuten von der Jägerhofstraße befindet sich der Malkasten, eine Künstlervereinigung, in der sich Studenten, so auch Lars Hertervig, und Akademiemitglieder treffen, Anliegen der Kunst und Künstler zu besprechen, und sich gesellig unterhalten. Der Künstlerverein Malkasten wurde am 06.08.1848 gegründet und hat auch heute noch seinen Sitz in der Jacobistraße 6a im Düsseldorfer Stadtteil Pempelfort. Hier in Düsseldorf verliebt sich Lars Hertervig in Helene, die 15-jährige Tochter des Hauses Winckelmann und entwickelt zunehmend erotomane Züge, die dazu führen, dass Helenes Onkel und Mutter ihm fristlos das angemietete Zimmer kündigen und ihn unter Hinzurufen der Polizei des Hauses verweisen. Lars Hertervig oszilliert nun obdach- und orientierungslos zwischen Malkasten und Jägerhofstraße hin und her und entwickelt – eindrucksvoll in Form eines inneren Monologs wiedergegeben – eine psychische Krise mit einer Vielzahl psychopathologischer Symptome, die aktuell am ehesten im Sinne einer akuten polymorphen psychotischen Störung oder auch einer beginnenden paranoiden Schizophrenie gedeutet werden würden. Der Autor macht dabei den Leser mit der anfänglich strikten Beschränkung auf das subjektive Erleben erst zum Phänomenologen, der den Sprung vom Objektiven in das Subjektiv-Erlebnishafte nachvollziehen muss, dann zum Psychopathologen, der wie in der phänomenologischen Psychopathologie von Karl Jaspers oder in deren Weiterentwicklung als deskriptiv-analytische Methode von Kurt Schneider die im Bewusstsein gegebenen psychischen Phänomene statisch erfassen muss, um dann den Zusammenhang des Seelischen genetisch zu verstehen und abstrahierend die unscharf begrenzten Phänomene in begriffliche Grenzen zu fassen. Vor dieser Aufgabe steht auch der Leser, wenn er Hertervigs Erleben immer wieder differenzieren muss in intrapsychisch Induziertes (wie bei der Halluzination oder dem Wahn) oder auf die reale Welt Bezogenes (wie bei der Liebesbeziehung), und wenn er vor diesem Hintergrund wie der Psychopathologe das Erleben verstehen und begrifflich fassen muss.

Zunächst ist da die erwähnte erotomane Idee, Helene und er liebten einander, die mit Streitsucht und Stalking sowie dem wie ein Eifersuchtswahn anmutenden Gedanken einhergeht, Helene und ihr Onkel könnten eine inzestuöse sexuelle Beziehung führen („*Du willst, daß dein Onkel deine Brüste anfasst, und niemand soll davon wissen. … Ich weiß das. Ich weiß, dass Dein Onkel dich angefasst hat …*“). Der Schlüssel zum Verstehen dieses psychopathologischen Syndroms ist immer der Beginn, handelt es sich um einen primären Wahn oder ist der eingeengte Affekt primär, aus dem sich ein sekundärer wahnhafter Überbau ableiten lässt. Hier lässt uns die phänomenologische Methode im Stich: Psychopathologisch ist Hertervig sicher affektiv eingeengt auf erotische Gefühle. Aber ist es einfache Verliebtheit, emotionale Begleitreaktion auf einen Liebeswahn, wonach er sie in sich verliebt wähnt, oder ist der eingeengte Affekt bereits Folge eines primären Wahnes, in dem er die Geliebte imaginiert oder wahnhaft verkennt (wie es sogar manche seiner Behandler annahmen).

Andere Textpassagen über Hertervigs Aufenthalte im Malkasten lassen Beziehungsideen im Sinne der einfachen Eigenbeziehung vermuten, vielleicht auch sekundär zu Halluziniertem („*er schaut sie an, sie nicken einander zu, … er will angeblich ein Maler sein, aber er ist ein schlechter Maler, er kann nicht malen, er bildet sich nur ein, dass er malen kann, aber alle wissen, dass er nur malen will … alle wissen das, sogar Gude weiß das, alle …*“; „*… die sitzen im Malkasten und reden über mich, oder hat er andere gemeint? Waren es die Leute im Malkasten, die über mich geredet haben? Oder andere Leute? Und die sitzen im Malkasten und reden über mich*“).

Weiterhin erlebt Hertervig offenkundig optische Halluzinationen („*Wieder kommen die schwarzen und weißen Tücher auf mich zu. Ich starre die schwarzen und weißen Tücher an.*“; „*Und ich sehe Helene mitten im Raum stehen bleiben und sie lächelt mir zu*“), akustische Halluzinationen, d. h. Stimmen, mit denen er kommuniziert („*Und dann ist deine Stimme in meinem Ohr, eine Stimme, die auf mich zukommt und sich um mich herumlegt“; „Und mein Vater ruft Lars Kommst du jetzt, Lars? Ja, ich komme, sage ich. Mit wem sprichst du?, fragst du.*“; „*Mit wem sprichst du?, fragt der, der neben mir sitzt. Also, jetzt musst du aber mal aufhören, mit Leuten zu reden, die gar nicht da sind, sagt der, der neben mir sitzt.*“), sowie taktile Halluzinationen („*hinter mir steht sie so schön und wie ein Engel … und dann legt Helene mir die Hände auf die Schultern …*“).

Als einfache Wahrnehmungsveränderungen (Metamorphopsie, Makropsie, Nahsehen) mit Übergängen zur Derealisation und zu optischen Halluzinationen können Passagen wie diese gedeutet werden: „*Ich sehe, dass dein Grinsen größer wird … dein Grinsen darf nicht so groß werden, dass es anfängt, sich von selber zu bewegen, es darf nicht auf mich zukommen … aber du wirst zu deinem Grinsen. Und ich nehme die Hände von den Augen weg und ich sehe dein Grinsen groß mitten im Zimmer stehen, dein Grinsen füllt das Zimmer fast ganz aus.*“.

Formalgedankliche Störungen erreichen nur manchmal den Grad der Zerfahrenheit als Faseln mit beziehungslos nebeneinanderstehenden Gedanken oder Gedankeninterferenzen („*Weißt du, dass die Kreuzotter sich schlängelt, sage ich. … Und ich bin Maler, ausgebildeter Maler. … Und mein Vater hat nämlich Pflaumen gepflückt. … Und meine Schwester geht durch die Straßen von Stavanger*“), sind über weite Strecken aber durch verbale Stereotypien geprägt mit zwangsähnlichen Perseverationen bestimmter Bewusstseinsinhalte respektive Verbigerationen/Iterationen (z. B. „*Und heute bin ich diese Straße ja auch entlanggegangen, dieselbe Straße hinunter. Ich bin dieselbe Straße hinaufgegangen. Ich gehe die Straße hinunter. Ich bin heute schon einmal diese Straße entlanggegangen. Ich bin die Straße hinuntergegangen. Ich bin diese Straße entlanggegangen, heute bin ich schon einmal diese Straße hinuntergegangen, und dann, etwas später, bin ich die Straße wieder hinaufgegangen. Ich gehe die Straße entlang.*“). Diese erheblichen, sich durch den gesamten Roman ziehenden und von Jon Fosse sprachlich eindringlich wiedergegebenen formalgedanklichen Störungen werden von manchem Leser geradezu als Stilmittel wahrgenommen, was bei Durchsicht der Rezensionen auf einer Internetplattform deutlich wird: „*Melancholie leidet an einer Krankheit …: ewige Wiederholungen. Es ist erstaunlich, wie man einen derart umfangreichen Roman mit so wenigen verschiedenen Wörtern füllen kann. … Vielleicht ist das Buch ein Versuch, dieses Irrewerden durch einen Blick in einen verwirrten, in sich selbst gefangenen Geist zu erklären. Nach längerer Lektüre fragt man sich als Leser jedoch selbst, ob der eigene Kopf noch klar ist.*“ oder „*Wieder, wieder und wieder wiederholen sich bei ihm die Wortfetzen, drehen, drehen und drehen sich Gedanken und Gefühle im Kreis. Anders als bei den Dramen wird die Lektüre dadurch bei diesem Roman etwas anstrengend.*“ [4].

Das nächste Kapitel des Romans berichtet am „*Morgen, Weihnachten, 1856*“ aus der „*Irrenanstalt Gaustad in Christiania*“, in die Lars Hertervig nun offenkundig eingewiesen wurde („*Ich liege im sechsten Bett … jetzt bin ich in der Irrenanstalt Gaustad, ich bin verrückt …*“). Das Gaustad Hospital (Gaustad sykehus), Oslo, Norwegen, wurde 1855, als Oslo noch Christiania hieß, als erstes psychiatrisches Krankenhaus in Norwegen eröffnet, dessen Direktor von 1855 bis 1882 Dr. Ole Rømer Aagaard Sandberg war [7]. Von diesem „*Doktor Sandberg*“ ist auch im Roman als Lars Hertervigs behandelndem Psychiater die Rede. Neben einer zunehmenden paranoid-halluzinatorischen Symptomatik tritt bei der literarischen Figur Lars Hertervig nun auch eine ausgeprägt gereizte bis fremdaggressive Komponente hinzu („*Verfluchte Scheißweiber, sage ich … Alle Weiber sind Huren, sage ich … Ich erschieße alle Weiber einzeln, sage ich … Ich bringe alle Weiber um, sage ich … Helene ist eine verfluchte Hure … Fotze, Fotze, sage ich …*“). Als ätiologisch verantwortlich für die psychische Dekompensation wird von Hertervig selbst bzw. von Dr. Sandberg vermutet: „*Ich habe zu viel Landschaft in starkem Sonnenlicht gesehen, darum bin ich verrückt geworden …*“ und „*ich höre Doktor Sandberg sagen, man kann meine Krankheit höchstwahrscheinlich dadurch erklären, dass ich mich da unten zwischen den Beinen angefasst habe.*“. Auf eine spezifische Therapie wird, abgesehen von arbeitstherapeutischen Ansätzen („*Ich soll gesund werden, … weil ich Schnee schippe*“), im Roman nicht eingegangen.

In der Tat ist historisch belegt, dass Lars Hertervig an einer sich während seiner Ausbildung an der Kunstakademie in Düsseldorf wohl erstmals manifestierenden psychischen Erkrankung litt und im Alter von 26 Jahren in der psychiatrischen Einrichtung Gaustad in Oslo, vormals Christiania, von Dr. Ole Sandberg behandelt wurde. Als therapeutische Elemente kamen nach kunsthistorischen Recherchen wohl „zweimal pro Woche heiße Bäder und kalte Duschen, fünf Tabletten für die Nacht und vier Blutegel im Nacken“ zum Einsatz [1]. Die damals gestellte Diagnose lautete zunächst „Melancholie“, wurde im Verlauf aber in „Dementia praecox“ abgeändert. Im April 1858 wurde Lars Hertervig „ungeheilt“ aus Gaustad entlassen [1, 8, 9].

Den zweiten Teil des Romans, „Melancholie II“, erzählt Jon Fosse aus Sicht Olines, der fiktiven Schwester Lars Hertervigs. Er kontrastiert damit die phänomenologische Methode des ersten Teiles, die die im Subjektiven verbleibenden Phänomene erfasst, mit der anderen möglichen psychopathologischen Methode, die sich auf die objektiv beobachtbaren und erfragten Begebenheiten beschränkt. Entsprechend steht dem reichen Innenleben des ersten Teiles im zweiten Teil eine im Verhalten beschränkte Person gegenüber. Oline schildert zunächst in einer Rückblende Hertervigs Kindheit und Jugend, in der sie bereits psychische Auffälligkeiten beim Bruder beobachtet haben will („*… sie sieht den Lars am Küchentisch sitzen und er starrt auf den Tisch und dann sieht es aus, als würde sein Blick sich an etwas festhaken, und dann hört sie, wie er anfängt, mit den Füßen zu stampfen, und … dann sehe ich es um seine Augen herum zucken …, er lässt sich einfach nicht weg rücken von der Stelle, an der er festgehakt ist, … und ich sehe, wie der Lars sich sozusagen losreißt, mit aller Kraft …*“; „*Diese Wut, die ihn manchmal packt. … Und dann wie er weint, manchmal weint er plötzlich los, sagt er. Und genauso plötzlich packt ihn diese Wut, dass er den Leuten fast an den Kragen will“; „Ich werde fast alle Maler umbringen, sagt er … er hat die Axt in all seiner großen Kraft auf das Holzstück geschwungen und in zwei Stücke gespalten … jetzt bist du hin, hat er gesagt.*“).

In einem Zeitsprung in das Jahr 1902 berichtet Oline von den letzten Lebensmonaten Lars Hertervigs in der Stadt Stavanger in Norwegen, wo der historische Lars Hertervig seit seinem siebten Lebensjahr in einem heute noch zu besichtigenden kleinen Haus in der Rosenberggaten 38 gelebt hatte und in die er auch im Roman nach seinem Aufenthalt in der „Irrenanstalt Gaustad“ zurückgekehrt war. Im höheren Lebensalter zog Lars Hertervig dann historisch belegt zunächst in das Haus seiner Schwägerin in der Rosenberggaten 26, anschließend in ein Armenhaus [5, 6]. Die literarische Schwester Oline beschreibt ein in diese letzten Lebensmonate fallendes eher depressives, angesichts der beschriebenen Harn- und Stuhlinkontinenz ggf. auch organisch anmutendes Bild: „… *und dann noch dieser wunderliche Bruder, der sicher ein großer Maler hätte sein können, und die schönsten Bilder hat er ja gemalt, aber gegen Ende zu hat er fast nur noch gekritzelt …“; „… früher ist er gern durch die Straßen von Stavanger gegangen, aber jetzt, jetzt will er das einfach nicht mehr, jetzt würde er am liebsten überhaupt nicht mehr rausgehen … Denn sie haben den Lars zwar in die Irrenanstalt Gaustad geschickt, damit er wieder gesund wird, aber seit er wieder zu Hause ist, will er gar nichts mehr tun …*“; „*der Lars hat weder das Wasser halten können noch das andere, und dann haben sie ihn oben ins Armenhaus gebracht und zu den anderen gelegt …“; „Ich bin ihn ja noch besuchen gegangen, als er im Sterben lag da oben unterm Dach im Armenhaus … und der Lars schaut ganz kurz zu mir her und ich sehe das schwere schwarze Licht in seinen Augen …*“.

Der historische Lars Hertervig starb am 06.01.1902 vermutlich an einem Magenkarzinom [1] und wurde auf dem Lagaard Cemetery in Stavanger, Norwegen, beerdigt. Eine Statue auf dem „Lars Hertervig Platz“ in Stavanger erinnert an den großen Naturmaler.

Die von Fosse beschriebenen Phänomene können Zeitlosigkeit beanspruchen als eine mögliche invariante Struktur menschlichen Erlebens, zeitlich und kulturell variant ist dagegen der wissenschaftliche Diskurs, der die Phänomene in Klassifikationssysteme einzuordnen versucht. Dem Autor gelingt es, unter Anwendung psychiatrischer Methoden die Dynamik eines reichen, sich möglicherweise auch in seiner Kunst manifestierenden Innenlebens einem monotonen, stereotypen Dasein gegenüberzustellen, ohne dass ihn die Diagnose einer Krankheit interessierte, anders als der Psychiater, der die psychopathologische Vielfalt begrifflich fassen will. In der psychiatrischen Rezeptionsgeschichte der biografisch, (kunst‑)historisch und psychiatrisch bekannten Daten und Fakten argumentieren die norwegischen Psychiater Øystein Førre und Svein Haugsgjerd [2], dass bei Lars Hertervig keine Schizophrenie, sondern eine „vorübergehende reaktive Psychose“ vorgelegen haben muss – vorübergehend u. a. deshalb, weil Lars Hertervig nach seiner Entlassung aus der psychiatrischen Klinik Gaustad 1858 keineswegs – wie seine fiktive Schwester Oline im Roman behauptet – „*gegen Ende zu … fast nur noch gekritzelt*“, sondern großartige Werke geschaffen hat, so z. B. im Jahr 1867 das Landschaftsbild „Die Insel Borgøya“, das im Nationalmuseum Oslo zu besichtigen ist und als Titelbild des bei Kindler verlegten Romans „Melancholie“ von Jon Fosse gewählt wurde (Abb. 1). Auch die norwegischen Mediziner Øivind Torkildsen und Elisabeth Farbu erkennen in Lars Hertervigs Biografie keinen künstlerischen, wohl aber einen sozialen Bruch nach der oben beschriebenen psychotischen Episode mit Aufenthalt in Gaustad und diskutieren als mögliche Diagnosen eine „reaktive Psychose“ vs. eine paranoide Schizophrenie im Sinne der historischen „Dementia praecox“ [11]. Nils Retterstøl, ehemals Professor für Psychiatrie an der Universität Oslo und Direktor der Psychiatrischen Klinik Gaustad von 1973 bis 1994, argumentiert ebenfalls eher in Richtung einer psychodynamisch-biografisch begründeten „reaktiven Psychose“ bzw. einer schizoaffektiven Störung mit einer gewissen Periodizität und günstigerem Verlauf als einer Schizophrenie im eigentlichen Sinne [10]. Gegen eine „akute vorübergehende psychotische Störung“ nach Diagnosekriterien des ICD-10 – hier am ehesten eine „akute polymorphe psychotische Störung mit Symptomen einer Schizophrenie“ (F23.1) oder eine „akute schizophreniforme psychotische Störung“ (F23.2) – bzw. eine „kurze psychotische Störung“ („brief psychotic disorder“; 298.8) nach DSM‑5 spricht aus heutiger Sicht allerdings die deutlich länger als ein bis maximal 3 Monate anhaltende Symptomatik. In der Wernicke-Kleist-Leonhard-Klassifikation schließlich, um die Relativität aller Diagnosen zu untermauern, wäre es eine affektvolle Paraphrenie mit der affektiven Verankerung der Wahnideen, dem Beginn mit einem Beziehungssyndrom und später Trugwahrnehmungen und Übergang zu einem gereizten Beziehungssyndrom und Veränderung der Affektivität in alle Richtungen.

**Figure 1:**
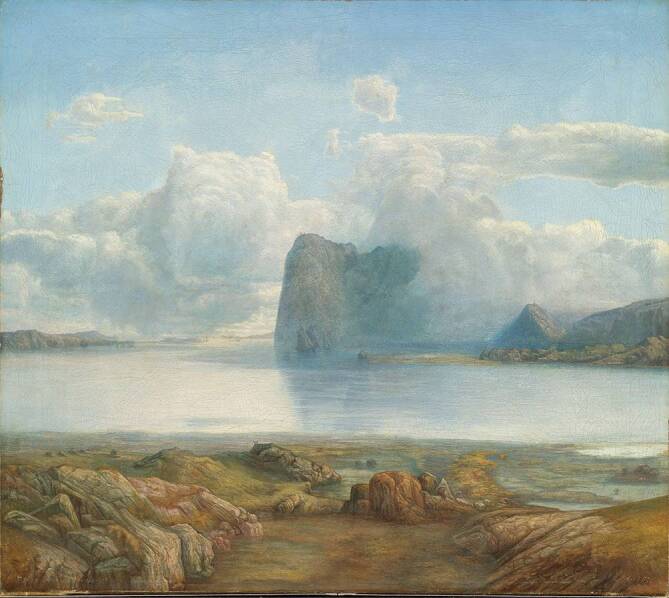

Der hier vorliegende Artikel hat in keiner Weise einen ferndiagnostischen Anspruch, sondern möchte der Leserschaft lediglich als Anregung dienen, sich Jon Fosses Roman „Melancholie“ nicht nur aus belletristischem, sondern auch psychiatrischem Interesse heraus zu nähern. In jedem Fall gewährt dieser Roman einen eindrucksvollen Einblick in die subjektive Wahrnehmung einer psychischen Erkrankung und deren Verschränkung mit höchster künstlerischer Schaffenskraft.

## Interessenkonflikt

K. Domschke war in den letzten drei Jahren Mitglied im „Steering Committee Neurosciences“ der Janssen-Cilag GmbH und hat Referentenhonorare von Janssen-Cilag GmbH erhalten. Sie ist Vorstandsmitglied der Deutschen Gesellschaft für Psychiatrie und Psychotherapie, Psychosomatik und Nervenheilkunde (DGPPN), des Anxiety Disorders Research Network (ADRN) des European College of Neuropsychopharmacology (ECNP), der Gesellschaft für Angstforschung (GAF) und des Freiburger Ausbildungsinstituts für Verhaltenstherapie (FAVT). D. Ebert gibt an, dass kein Interessenkonflikt besteht.
